# Study on path planning of mechanized harvesting of ratoon rice in the first season based on the capacitated arc routing problem model

**DOI:** 10.3389/fpls.2022.963307

**Published:** 2022-10-10

**Authors:** Guozhong Zhang, Chao Ji, Qing Wu, Haopeng Liu, Yong Zhou, Jianwei Fu

**Affiliations:** ^1^ College of Engineering, Huazhong Agricultural University, Wuhan, China; ^2^ Key Laboratory of Agricultural Equipment in Mid-Lower Yangtze River, Ministry of Agriculture and Rural Affairs, Wuhan, China

**Keywords:** ratoon rice, first season harvester, capacitated arc routing problem, route planning, genetic alghorithm

## Abstract

The stubble crushing caused by the harvester during the first season of ratoon rice harvesting will directly affect the grain yield of the ratoon season. In this work, a harvester path planning method for quadrilateral fields to address the harvester driving path problem of the first season of ratoon rice mechanized harvesting is proposed. This research first analyzes the operational characteristics and requirements of ratoon rice first-season mechanized harvesting, and then models the mechanized harvesting process of ratoon rice in the first season as a capacitated arc routing problem (CARP) considering the fact that the harvester cannot complete the full-coverage harvesting operation at one time due to the limitation of grain bin volume. The genetic algorithm (GA) with strong global search capability is used to solve it, and the selection and variation links of the algorithm are improved. The path planning method proposed in this article can dynamically find the optimal harvester travel route according to the specific conditions of the field and the parameters of harvester implements. The simulation test shows that the CARP method performs better in terms of harvesting path length and crushed area compared to the conventional rectangular detour and foldback reciprocating harvesting paths. The degree of optimization of this method is influenced by various factors such as the width of the cutting table, the turning radius of the harvester, and the size of the grain bin capacity. This research provides a more efficient and flexible path planning method to improve the efficiency of ratoon rice first-season mechanized harvesting operations and optimize the harvester’s operating path, which can well meet the operational requirements.

## 1 Introduction

The driving path of the harvester in the traditional harvesting operation is planned by the harvester according to the operator’s experience and combined with the grain transfer location of the field independently, but this is not always the path with the best overall efficiency (Bochtis et al., 2014). In the process of agricultural operations, almost all operations essentially involve the movement of vehicles, of which the agricultural machinery operation path planning that combines the quality, time, and cost of field operations is an important part of future agricultural production intelligence (LI and LI, 2020). Efficient and reasonable path planning can reduce operating costs, improve harvesting efficiency, increase farmers’ trust in new farm equipment, and promote the continued development of new technologies (Yajie et al., 2021). The driving path length of agricultural machinery in the process of operation is divided into the essential path length and non-essential path length, where the non-essential path corresponds to the time that is non-essential working time. Reasonable path planning can effectively reduce the length of the non-essential path during agricultural machinery operations, shorten the operating time (Bakhtiari et al., 2013), and improve the efficiency of field operations (Jensen et al., 2015). Therefore, it is necessary to plan and design the path of travel during the operation of agricultural machinery to ensure satisfactory results of the operation process.

To optimize the path problem for field vehicle operations, researchers have used many different research methods, mainly dealing with the coverage path planning problem and the routing problem. In some studies, these two methods are combined.

In terms of coverage path planning problem research, Bochtis (2008) proposed a *B*-pattern farm machinery operation model that divides farmland into multiple parallel, equal-width operation rows, transforming it into a travel quotient problem to optimize farm machinery operation paths. In the same year, it was transformed into a binary integer planning problem, and a parallel traversal sequence finding algorithm was proposed, which can reduce the unnecessary operational cost by more than 50% compared to the reciprocate operational paths. Jin and Tang (2010) proposed a geometric representation method with similar properties to the potential field, and combined this method with a path planning algorithm to solve the optimal coverage path by decomposing the farmland into sub-regions and separately determining the direction of farm machinery operations within the sub-regions, and establishing a geometric model of the farmland. Hameed et al. (2011) calculated the optimal operating travel direction of farm machinery based on the geometry of farmland to optimize the operating path of farm machinery. Bakhtiari et al. (2013) proposed the use of an ant colony optimization method to generate a combine harvester field mulching scheme with a non-working distance saving of about 18% to 43% compared to the conventional scheme. Conesa-Muñoz et al. (2016) use simulated annealing algorithms to calculate cover crop trajectories for vehicles with different characteristics. Furthermore, the authors propose to integrate the Mix-opt operator into the algorithm to optimize the travel path of multiple farm machines working together (Conesa-Mu N Oz et al., 2016). Oksanen and Visala (2009) proposed the “trapezoidal decomposition method” and “cell partitioning method” to solve the problem of full coverage of farm machinery operations under complex field boundary conditions, and used the local optimal strategy to solve the problem continuously downward until the entire field was covered. Yao et al. (2019) proposed a conflict-free cooperative operation path optimization algorithm for multiple combine harvesters, which effectively improves the field operation by avoiding conflicts, and improved the simulated annealing algorithm based on Doppler and greedy strategy for large-scale farming operations, which led to some improvement in the solution performance of the algorithm (Jingfa et al., 2020). Utamima et al. (2019) proposed an evolutionary hybrid neighborhood search method to solve the optimal path problem in the field of agricultural machinery.

In the study of routing of path planning problems, Bochtis and Sørensen (2009) defined the agricultural machine operation problem as a vehicle routing problem (VRP). For solving the optimal operation path for agricultural fields, the field coverage is represented as a traversal of the weighted graph, and it is shown that the problem of finding the optimal path is equivalent to finding the shortest traversal in the weighted graph. Reid (2004) proposed a method combining VRP and the least-cost network flow problem in order to determine the optimal coverage path for combines and the feasible location for grain transfer between combines and tractors. The expansion of the VRP problem gives rise to the arc routing problem with capacity constraints (CARP). The goal of the CARP problem is to determine one complete trip of the vehicle from the depot until it returns to the depot, and to incur as little cost as possible for the entire trip. The CARP problem was introduced by Golden et al. (1983), and was shown to be an *NP*-hard problem by Corberan and Laporte (2015). Regarding the CARP problem, many research scholars have carried out research work; Seyyedhasani et al. (2019) conducted mathematical modeling with the CARP problem for the case of multiple farm machines involved in the operation, and proposed an improved Clark–Wright algorithm and a taboo search algorithm to optimize the operation path of multiple farm machines, resulting in a 32% reduction in operation completion time. Pitakaso and Sethanan (2019) modeled the CARP problem on the sugarcane harvesting problem and used the adaptive large neighborhood search (ALNS) algorithm to solve the problem of maximizing the area of sugarcane harvested under the condition that only a certain number of sugarcane harvesters are available. Numerous metaheuristics such as Genetic algorithm (GA), Taboo search (TS) algorithm, Adaptive large neighborhood search (ALNS) algorithm, and Ant colony optimization (ACO) algorithm can be used to solve the CARP problem (Corberan and Laporte, 2015). In these intelligent algorithms, GA has better stability and constructivity in solving problems with search properties (Ahmed et al., 2022). The efficient solving speed of the metaheuristic algorithm can give high-quality path results quickly, but the characteristics of the solving algorithm vary, and the problem of the structure of the solving algorithm under multi-conditional constraints has to be solved.

Along with the development of social science and technology, agricultural equipment is developing in the direction of intelligence and precision (LI and LI, 2020). Improving grain yield per unit area has become a key element of future research in the field of agriculture. Compared with ordinary rice, ratoon rice has the advantages of saving labor and seeds, and vigorously promoting grain output and rural income, and it is being promoted in the middle and lower reaches of Yangtze River (Fe and Shaobing, 2018). It is different from normal rice in that only one full cover harvest is carried out after maturity. Ratoon rice harvest is divided into the first season and the regenerated season harvest. Regenerated season harvesting of ratoon rice is of the same type as ordinary rice harvesting, and most studies in this direction have been conducted with the driving path length as a single objective. However, the first-season harvesting of ratoon rice is different and needs to meet more complex harvesting requirements. Ratoon rice needs to take into full consideration the crushing damage caused by the harvester driving on the stubble; thus, the crushing area generated by the harvesting operation is also an important evaluation aspect. The path planning of the first harvest of ratoon rice is not only closely related to the yield of the first-season harvest, but also a reliable guarantee of getting high yield in the regenerated season. The travel path obtained by reasonable planning can obtain a lower stubble breaking rate on top of reducing the crushed area, resulting in a significant increase in the regeneration rate of regenerated rice and the yield of the regeneration season.

In order to solve the path problem of the harvester during the harvesting of the first season of ratoon rice, this study integrates the volume of the grain bin of the harvester and the location of the grain unloading point, and introduces the CARP problem, which is adapted to this problem, as an optimization tool to solve the path planning model by improving the GA with good constructivity and faster solution speed to obtain the final driving path. From the results, this research effectively shortens and reduces the driving path length and crushing area of the ratoon rice harvester during operation, improving the overall efficiency of the harvesting operation to help increase the harvest in the regenerated season.

The remainder of the article is organized as follows. *Section 2* gives the requirements and characteristics of the first-season machine harvesting operation of ratoon rice and the transformed CARP model with the solution algorithm. Conducting example and simulation tests and investigating the factors that affect the effectiveness of planning are given in *Section 3*. Finally, conclusion and the future research directions that can be studied in depth are presented in *Section 4*.

## 2 Materials and methods

### 2.1 Requirements and characteristics of ratoon rice first-season harvesting operation

**Figure 1:**
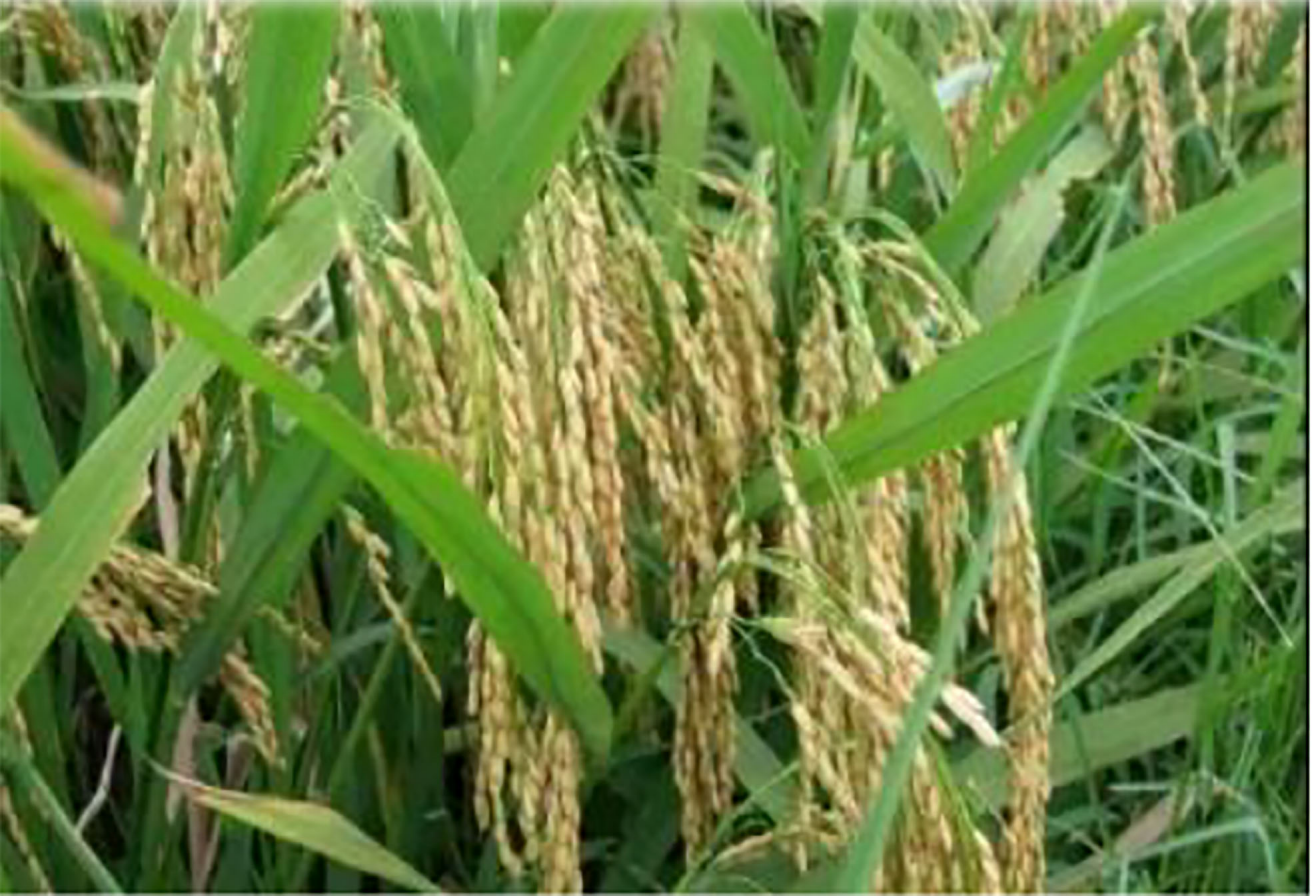
Ratoon rice.

**Figure 2:**
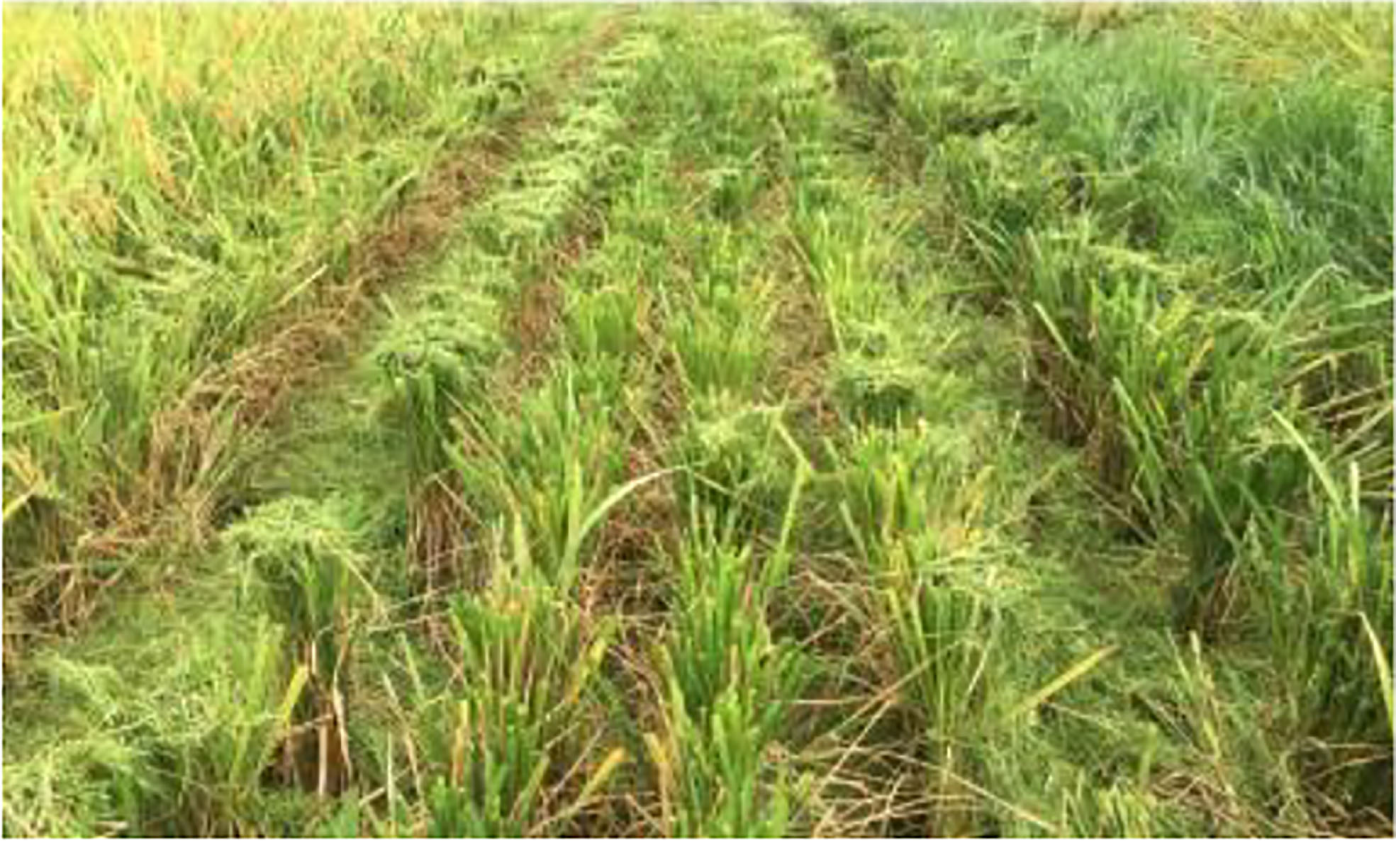
Rice stubble after first-season harvest.

Ratoon rice (**Figure 1**) has been popularized in the middle and lower reaches of the Yangtze River because of its unique cultivation and management measures that allow the once-harvested stubble to continue to sprout into ears, which meets the requirements of modern agriculture to increase food and income (Xu et al., 2015). The yield of the second season of ratoon rice depends on the crushing of rice stubble by the harvester in the first season. Large crushing area or heavy crushing degree on rice stubble will lead to reduced yields in the second season (**Figure 2**). Therefore, the main agronomic requirements for the first-season harvest of ratoon rice are less crushed area and lighter crushed degree.

With single-transplant rice seedlings, the ratoon rice can be harvested twice in July and November. In terms of yield, the first harvest yields significantly more than that in the regenerated season (Ling, 2022). According to the agronomic requirements of the first-season mechanized harvesting of ratoon rice, if the grain bin capacity of the ratoon rice harvester is huge, the harvester will bring about serious crushing of the rice stubble in the rest of the harvest path after loading a large number of rice ears, resulting in difficulty for the stubble to sprout into ears again, which will have a serious impact on the grain yield of the regenerated season. Therefore, the grain bin capacity of the ratoon rice first-season harvester is generally small. Moreover, if the farmland to be harvested is large, several grain unloading sessions are required during the operation to complete the full-coverage harvest of the field. Combined with minimizing the unnecessary harvest path length during the operation of the harvester and improving the operation efficiency, it undoubtedly presents a new challenge to the harvest path planning of the harvester, which also means that the research on the driving path planning of the harvester for the first harvest of ratoon rice is of great importance.

### 2.2 Ratoon rice first-season harvester turning mode and distance calculation in field

#### 2.2.1 Dual-channel feed ratoon rice first-season harvester

**Figure 3:**
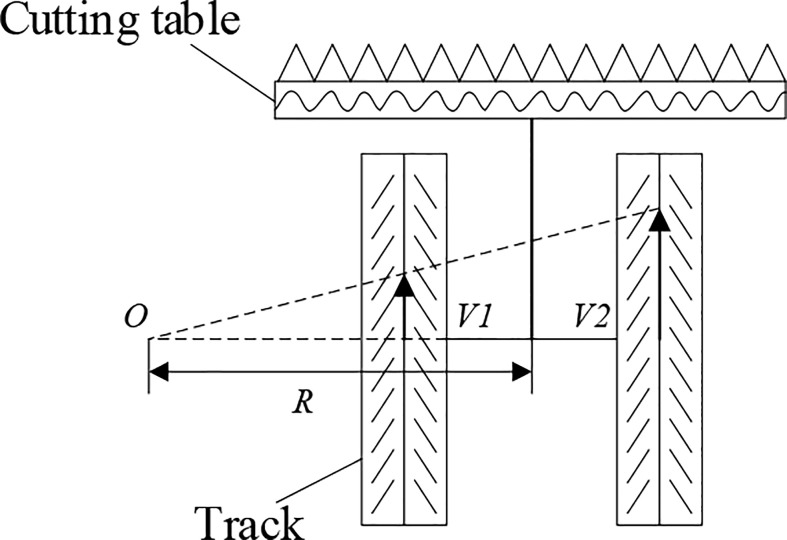
Turning radius of tracked chassis.

It is necessary to irrigate the farmland in the middle and lower reaches of Yangtze River frequently. This results in very obvious boundaries between fields and more obstacles that are not easy to pass, such as field ridges, ditches, and slope banks. In order to determine the passing performance of the harvester, a tracked chassis is generally used. For the first-season harvest of ratoon rice, the research team of the authors modeled the milling of crawler chassis during the first-season mechanized harvesting of ratoon rice and analyzed in detail the relationship between each dimensional parameter of the harvester and the crushing rate. The turning radius principle of the harvester is shown in **Figure 3**. The turning action of the tracked chassis relies on the different winding speed and direction of travel of the tracks on both sides to complete. Assuming that the effects of slip and deflection caused by the soil are not considered, the tracked chassis is divided into left and right turns according to the different travel speeds of the left and right tracks. When V2 > V1 or V2< V1, the tracked chassis will turn to the side with less speed, and the center of rotation O will change with the ratio of track speed on both sides; meanwhile, in order to reduce the crushing rate, the tracked harvester gives priority to a larger turning radius (Lei et al., 2017). When the speed of both tracks is in opposite directions, the center of rotation of the machine falls between the two tracks and the chassis moves in a circle around the center of rotation. In the operation, the harvester generally turns while harvesting, and adopts dynamic variable speeds for turning operation. The size of the turning radius also varies with the forward speed of the tracks on both sides, and the turning radius is affected by the slip rate between the tracks and the soil. Thus, the actual turning radius value is larger than the theoretical turning radius value eventually. In this research, the turning radius is assumed to be a constant value when conducting simulation tests, but multiple sets of different values will be taken for verification.

**Figure 4:**
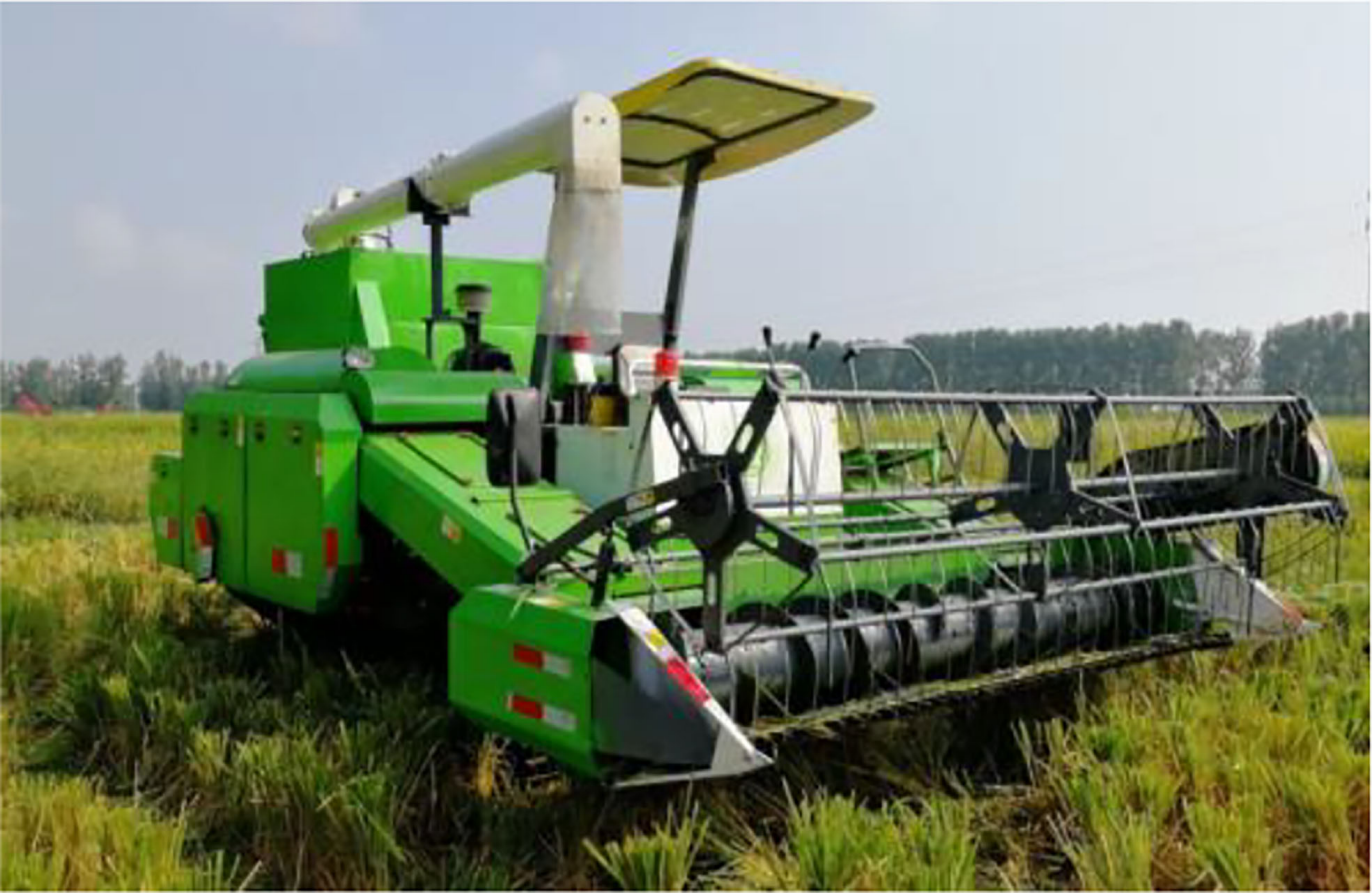
Dual-channel rice first-season harvester.

Based on the existing research foundation of our team, the dual-channel feed ratoon rice first-season harvester (**Figure 4**) is able to better meet the low crushing rate requirement during the ratoon rice first-season mechanized harvesting, and also has improved field harvesting efficiency compared to the conventional harvester. More importantly, the yield increased by 23.9% in the second crop harvested by the double-channel feeding harvester compared to the conventional harvester (Fu et al., 2020). This article refers to the specific parameters of this model for calculation when testing examples. The parameters related to the dual-channel feed ratoon rice first-season harvester are shown in **Table 1**.

**Table 1:** Specific parameters of double-channel feed ratoon rice first-season harvester.

Parameters	Value
Whole machine size (length × width × height) (mm)	5,260 × 3,340 × 2,850
Whole machine quality (kg)	3,250
Feeding volume (kg·s^−1^)	≥4.0
Cutting table width (mm)	3,000
Track width (mm)	400
Grain bin volume (L)	1,500
Minimum turning radius (m)	4

#### 2.2.2 Harvester turning mode

Harvester crushing of field consists of two parts: straight and turning. The straight crush is determined by the track width of the harvester, but the path length can be reduced by planning. What cannot be ignored is the straight-line portion crush of the stubble in the same direction, but the stubble recovers better after being crushed. However, the crush caused by the turn is devastating, causing damage to the stubble from the roots, and the stubble crushed by the turn will hardly sprout into ears again in general. Therefore, one of the goals of path planning is to reduce the number of turns during operations and to make turns with as large a turning radius as possible. When the harvester travels in the harvested area, it should follow the existing crushing track to reduce the crushing damage to the rice stubble.

**Figure 5:**
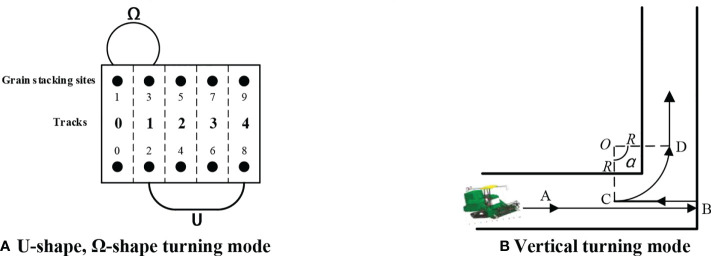
Turning mode **(A)** U-shape, Ω-shape turning mode **(B)** Vertical turning mode.

The types of harvester turns in the field are mainly divided into turns between harvest rows and turns at boundary corners (Zhou and He, 2021). Huang et al. (2014) proposed three common turning strategies for U-shape, Ω-shape, and T-shape (Fishtail type) for the common turning mode in production operation. In order to simplify the operation of agricultural machinery, this research uses both U-shape and Ω-shape turns between harvesting rows. Most importantly, this article proposes a new turning mode that reuses the driving trajectory of the harvester for boundary corners and where vertical turns are required, as shown in **Figure 5B**. After the harvester travels along the horizontal operation line to the boundary of the vertical operation line, it reverses a distance of one turning radius from the original road, and then performs a turning operation along the arc formed by the turning radius of the harvester, and smoothly transitions to the vertical operation line in the order indicated by the letters shown in the figure: $\underset{˙}{\text{A}}\underset{˙}{\text{B}}\underset{˙}{\text{C}}\text{D}$. This kind of turning strategy can effectively reuse the crushing traces already produced by the harvester in the process of traveling, avoiding new damage to the stubble in the harvested area and reducing the crushing loss. The three types of turns used in the research and the calculation of the turning distances are shown in **Figure 5**.

Take rectangular field as an example, after the harvester has finished working from row *i* to the next row *j*. The path lengths for U-shape and Ω-shape, respectively, are as follows:


(1) $$\begin{array}{c} D_{ij}^{\Omega }=2R_{0}\left(\pi -tan^{-1}\frac{n_{ij}\cdot W}{\sqrt{4R_{0}^{2}-n_{ij}^{2}W^{2}}}\right),\;i\neq j \end{array}$$

(2) $$D_{ij}^{U}=W\cdot n_{ij}+\;(\pi -2)R_{0,}i\;\neq j$$

where $D_{ij}^{\Omega }$ is the Ω-shape turning distance, m; $D_{ij}^{U}$ is the U-shape turning distance, m; *W* is the width of harvester cutting table, m; *R*_0_ is the minimum turning radius of harvester, m; *n*_*ij*_ is the turning interval between operating rows *n*_*ij*_=|*j*−*i*|, *j* ≠*i i* is the harvester end row number, and *j* is the harvester entry row number.

Harvester ground head turning distance length determination is as follows:


(3) $$\begin{array}{c} L_{min}\left(D_{ij}\right)=\{\begin{array}{c} D_{ij}^{\Omega }\;,\;n_{ij}<\frac{2R_{0}}{W}\\ D_{ij}^{U}\;,\;\;n_{ij}<\frac{2R_{0}}{W}\; \end{array},\;i\neq j \end{array}$$

where *L_min_* (*D_ij_*) is the turning distance at the head of the field, m.

The length of the turning path of the harvester at the boundary corners and where vertical turns are required is as follows:


(4) $$\begin{array}{c} D_{Z}=R_{0}\left(1+\frac{\alpha \pi }{180}\right) \end{array}$$

where *D*_*Z*_ is the distance of vertical turning, m; and *α* is the angle at which the turn was made.

#### 2.2.3 Construction of turning distance matrix

According to the boundary parameters of the field, the width of the harvester cutting table is used as the fixed width of the harvesting row for operating row division. In order to observe the differences in the number of turns brought by the long and short side branches of the field, this study compared rectangular fields with the same width and different lengths. The values of the long and wide sides of the field were taken from the general statistical rules of the field size parameters in the middle and lower reaches of the Yangtze River. Experimental data are shown in **Table 2** (*W* = 3).

**Table 2:** Differences in the number of turns in the long and short side branches of the field.

Field length × width (m)	Number of turns		Turning number difference
	Short side branch	Long side branch
40×60	14	20	6
40×80	14	27	13
40×100	14	34	20

As **Table 2** shows, compared with the longer side of the farmland as the basis for branching, the shorter side branches obtained fewer turns, and the difference in the number of turns became larger as the difference between the long and wide sides became larger. Therefore, this harvest path planning method designed in this research uses the shorter edge of the field as the basis for branching, and then generates the set of crop row codes *T*:


$$T=0,\;1,\;2,\;\cdots ,\;n\;\;\;\;n=\frac{l}{W}-1$$

where *l* is the length of field shorter edge, m.

After obtaining the operation crop row code *T*, this study assumes that the weight of grain between the operation rows is uniformly distributed at the stacking points at both ends of the operation crop row (**Figure 5A**). The harvesting of this operation crop row is transformed into the harvester passing through the two grain stacking points of the crop row in turn. Therefore, the set *N* of stacked point codes can be obtained from the crop row codes:


N=0, 1, 2, ⋯, 2n+1

In this research, let the even-numbered stacking points and the odd-numbered stacking points be distributed in pairs, as shown in **Figure 5**, and the distance traveled by the harvester between different crop rows is equal to the distance between two stacking points on the same side in different operation rows. The distance between stacking points is calculated as shown in **Table 3**, and the distance matrix between stacking points in the field is obtained as follows:


(5) $$\begin{array}{c} A=\left[\begin{array}{cccccc} 0 & L_{01} & a_{02} & 0 & \cdots & a_{0n}\\ L_{10} & 0 & 0 & a_{13} & \cdots & a_{1n}\\ a_{20} & 0 & \ddots & L_{23} & \cdots & a_{2n}\\ 0 & a_{31} & L_{32} & \ddots & \cdots & a_{3n}\\ \vdots & \vdots & \vdots & \vdots & \ddots & \vdots \\ a_{n0} & a_{n1} & a_{n2} & a_{n3} & \cdots & a_{nn} \end{array}\right] \end{array}$$

**Table 3:** Calculation method of distance between stacking points.

Crop row stacking point	Calculation method or value
0⇿1	*l*−2*.* *w w* is the width of field head turning area
0⇿2	Same side turn: if *W*_*i* *j*_ ≥ 2*R* -U-shape, else Ω-shape
0⇿3	Does not meet the rules of marching: 0
1⇿2	Does not meet the rules of marching: 0
1⇿3	Same side turn: if *W*_*i* *j*_ ≥ 2*R* -U-shape, else Ω-shape

where *a*_*nn*_=0 *a*_*ij*_=*a*_*ji*_ *L*_*ij*_=*L*_*ji*_=*Li*∈*Nj*∈*Ni*≠*j* The distance length value is set to 0 for the case that cannot be achieved by one turn between stacking points (including turns between the same stacking points), where *L* is the length of the fixed distance (constant) between two stacking points in the crop row.

### 2.3 Routing planning model based on CARP

#### 2.3.1 Field boundary treatment

Both turning operations during the first-season mechanized harvesting of ratoon rice are unique. Turning at the corner is limited by the shape of the field, which occurs only a limited number of times during the whole operation and has a limited grinding area, while the turning operation between the operation rows is the main reason for the grinding loss during the harvesting of the first season of ratoon rice. In the research on path planning for agricultural mechanized harvesting operations, most of them use manual harvesting of the turning area or harvesters around the field boundaries to obtain the turning area at the head of the field (Bochtis and Sørensen, 2009).

In order to reduce the losses caused by turning between crop rows and improve the overall smoothness of the harvesting operation, this research proposes a method of handling the turning area at the head of the field that combines the turning radius of the harvester with the volume limitation of the grain bin. The operation process is divided into two parts: turning area at the head of the field harvesting and operation row strip harvesting, where the width of the head turn area of the field is calculated as follows:


(6) $$\begin{array}{c} W_{t}=\frac{2R+W}{2W}\cdot W \end{array}$$

where *W_t_* is the width of field turning circle, m, and *R* is the turning radius of harvester, m.

**Figure 6:**
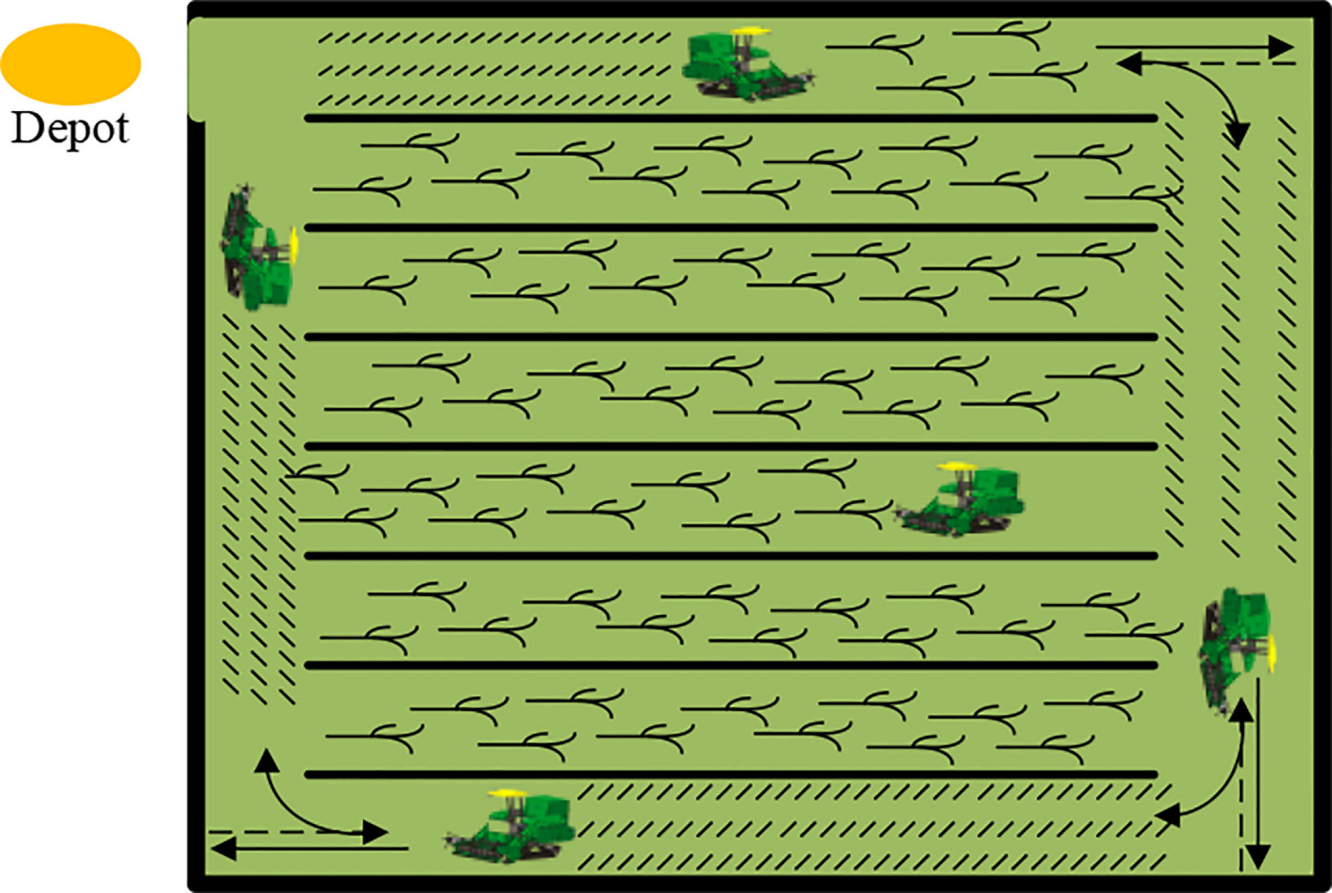
Field boundary treatment.

The harvesting strategy for the field head turn area is to first determine the maximum path length of the harvester for a single trip based on the relationship between the volume of the harvester’s grain bin and the weight of the grain in the unit area. When the sum of the field boundary length is greater than the maximum path length of the harvester for a single trip, the location of the entered operation crop row is selected according to the field boundary length, and the field boundary and the operation row are used to form the boundary of the sub-region for harvesting (**Figure 6**). When the sum of the field boundary lengths is less than the maximum path length of the harvester in a single trip, the harvester can harvest around the field boundary and unloads grain at the depot until the turnaround area of the field is completed.

The specific method for crop strip harvesting is to model the CARP for the set of unharvested crop rows. An improved GA is used to solve the harvesting sequence of the operating rows and guide the harvester to complete the harvesting operation.

#### 2.3.2 CARP

The capacitated arc routing problem (CARP) is generally described as an undirected graph *G (V*, *E)* with weighted connectivity, where *V* and *E* are denoted as the set of vertices and the set of arcs, respectively. Each arc (*E*_*i*_, *E*_*j*_ ) in the graph is a subset of *E* containing two attributes, demand *q*_*ij*_ and cost consumption *c*_*ij*_ from node *i* to node *j*. The demand and cost consumption are non-negative value, and the edge where the demand is greater than zero is called the “task edge”. In this research, the demand of arc is set as the sum of the weight of two stacking points in the same operation crop row, and the cost consumption is set as the distance between two stacking points. In the CARP, the location of the depot from which the vehicle departs and arrives is transformed into a grain unloading point (depot) in the field. There is only one depot in each field, and it is located where the harvester enters the field in this research. The maximum carrying capacity of the harvester *Q* ( *Q*≥*max{q*_*ij*_} , *i*, *j* ∈ *E* ) is the maximum weight that can be loaded in the grain bin of the harvester. CARP is defined in the first season of ratoon rice mechanized harvesting process as the harvester starts from the grain transfer point, harvests as many crop rows as possible before the grain bin is full, and only allows each crop row to be traversed once. Finally, the harvester returns to the depot for unloading operations.

After modeling according to the CARP, the optimization objective of the path planning for the first-season mechanized harvesting of ratoon rice is to find the path with the shortest distance traveled by the harvester after the harvester has traversed all the operating crop rows and satisfies the following constraints:

(1) Each path of the harvester starts from and eventually returns to the depot.
(2) Each operating crop row must be met in one and only one harvester travel route.
(3) The total weight of grain loaded in each route on which the harvester travels cannot exceed the grain bin capacity limit.
(4) The end of the previous operation line is the starting point of the next operation line during the operation of the harvester.
(5) Both the number of depots and the number of harvesters in the field to be harvested are assumed to be 1.

The mathematical description of the CARP and the associated functions refer to Golden who first formulated the problem, the objective, and constraint functions as follows (Golden and Wong, 1981):

Objective Function:


(7) $$\begin{array}{c} \min\sum_{i=1}^{n}\sum_{j=1}^{n}\sum_{p=1}^{k}c_{ij}x_{ij}^{p} \end{array}$$

Constraint Function:


(8) $$\{\begin{array}{l} \sum_{k=1}^{n}x_{ki}^{p}-\sum_{k=1}^{n}x_{ik}^{p}=0,\forall i=1,2,\cdots ,n;\forall p=1,2,\cdots ,k.\\ \sum_{p=1}^{k}\left(l_{ij}^{p}+l_{ji}^{p}\right)=\left\lceil \frac{q_{ij}}{W}\right\rceil ,\forall (i,j)\in E.\\ x_{ij}^{p}\geq l_{ij}^{p},\forall (i,j)\in E,\forall p=1,2,\cdots ,k.\\ \sum_{i=1}^{n}\sum_{j=1}^{n}q_{ij}l_{ij}^{p}\leq W,\forall p=1,2,\cdots ,k.\\ \sum_{i\in \tilde{Q}}\sum_{j\in \tilde{Q}}x_{ij}^{p}+n^{2}y_{1\tilde{q}}^{p}\leq \left|\tilde{Q}\right|-1\\ \sum_{i\in \tilde{Q}}\sum_{j\in \tilde{Q}}x_{ij}^{p}+y_{2\tilde{q}}^{p}\geq 1\\ y_{1\tilde{q}}^{p}+y_{2\tilde{q}}^{p}\leq 1\\ y_{1\tilde{q}}^{p},y_{2\tilde{q}}^{p}\in \{0,1\} \end{array}$$

where $\tilde{q}=1,\;\;\cdots ,\;k;\;\forall \tilde{Q}\in V,\;\left|\tilde{Q}\right|\geq 2$ $x_{ij}^{p},\;l_{ij}^{p}\in \left\{0,\;1\right\},\;\forall i=1,\;\;\cdots ,\;n;\forall j=1,\;\cdots ,\;n;\forall p=1,\;\cdots ,\;k.$

### 2.4 Improved genetic algorithm

As previously mentioned, CARP is an NP-hard problem. In the case of large instances, it is sometimes impossible to obtain the optimal solution in a short time by using exact algorithm, and only the high-quality solution that meets the requirements can be sought in a certain time (Corberan and Laporte, 2015). Therefore, the solution objective of this research is to find the forward route of the harvester with a shorter driving path length in a certain time. Compared with other algorithms, the GA is able to compare multiple individuals at the same time from the group perspective, effectively avoiding the disadvantage of easily falling into dead loop. In addition, the GA has strong stochastic search capability, outstanding scalability, and can be flexibly adjusted according to different problems and conditions.

GA is a meta-heuristic algorithm that simulates the process of inheritance and evolution of organisms in their natural environment. It is essentially a parallel, efficient, global search method that can automatically acquire and accumulate knowledge of the search space during the search process and adaptively control the search process to find the optimal solution. Similar to biological evolution in nature, population evolution follows the principle of “survival of the fittest”. Individual selection, heredity, variation, and other operations will occur in each generation of population. The adaptive individuals will survive, and the unsuitable individuals will be eliminated. Finally, a new population will be generated. Compared to traditional optimization algorithms, GA is a robust search algorithm that can be used to optimize complex systems with the following characteristics:

a) Convenient manipulation of genetic operators with the encoding of decision variables as arithmetic objects to simulate the genetics and evolution of organisms.
b) Using the fitness value as the search information, and using the fitness function to narrow the search and improve the search efficiency.
c) Multi-point parallel search for faster approximation of the global optimal solution.

**Figure 7:**
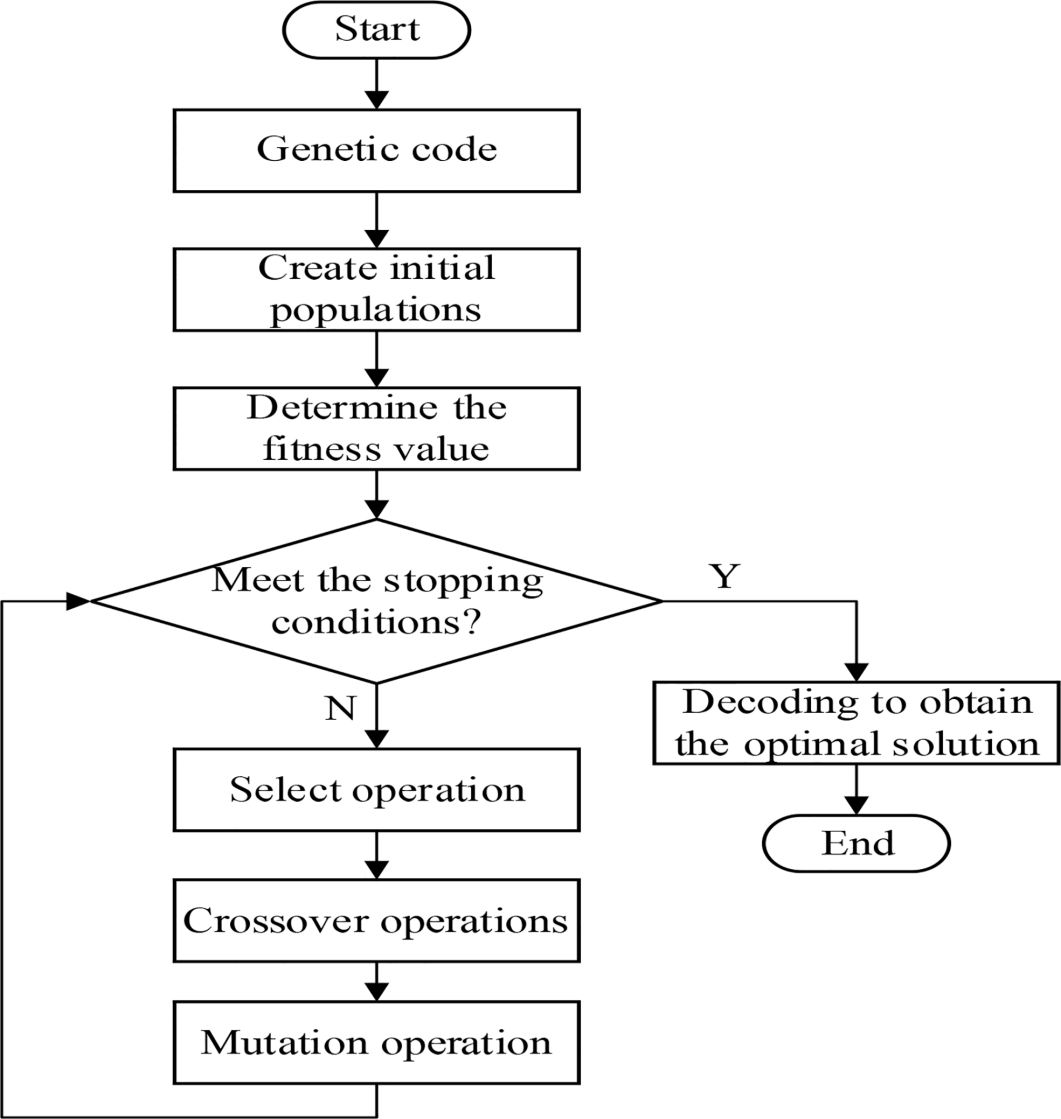
Genetic algorithm flowchart.

The flow of GA for optimal solution search is shown in **Figure 7**, and its process can be mainly described in six steps: encoding, initial population generation, fitness evaluation, selection, crossover, and variation, until the optimal solution is obtained (LIU et al., 2022).

There are two main improvements to GA in this study. Firstly, the optimal individual preservation strategy is used in the selection operation due to the speeding up of the algorithm search, which means that the individual with the largest fitness value in the population directly replaces the individual with the smallest fitness value, and no crossover and variation operations are performed between them. They are directly copied to the next generation and the fitness value is calculated again to prevent the crossover and variation operations from destroying the excellent solutions in the population. Secondly, the inverted variation method is used in the mutation operation, where two genes from the same individual are randomly selected if the current random probability value is less than probability parameters. Then, the middle part of the two is inverted, resulting in a new individual.

The termination condition of the algorithm is to exceed the set number of iterations and output the optimal solution obtained during the iteration.

## 3 Results and sensitivity analysis

The path planning method proposed in this article is tested with real field, obtained through the area calibration function of Google satellite maps, and the algorithm coded in Matlab R2018b and run on an Intel(R) Core (TM) i7-8550U CPU and 16 GB of RAM to solve the harvesting agricultural operations in the field.

In the traditional harvesting process, the harvesting path method is often adapted to the specific environment and conditions of the field itself, with the regular-shaped field using the rectangular detour harvesting method, while the irregular-shaped field is more popular with adjacent strips of the work “bulb-type” turning method (Nilsson and Zhou, 2020; Chengming et al., 2021). The following simulation tests were conducted to verify the optimization effect of the harvest path planning method proposed in this paper compared with the traditional harvesting method and the factors affecting the path planning effect.

### 3.1 Comparison of different path planning methods in regular-shaped fields

The location of the rule field was selected as a ratoon rice planting field in Siyumen Village, Wulin Town, Honghu City, Hubei Province, and the outline information of the simulation test plots is shown in **Table 4**. The ratoon rice variety is Fengliangyouxiang No. 1, with a first-season yield of 10,175.4 kg/hm^2^ (Fu et al., 2020).

**Table 4:** Simulation test regular field plot contour point information.

Point Name	Latitude (°)	Longitude (°)
0	29.86266	113.55088
1	29.86237	113.55122
2	29.86186	113.55065
3	29.86213	113.55031

**Figure 8:**
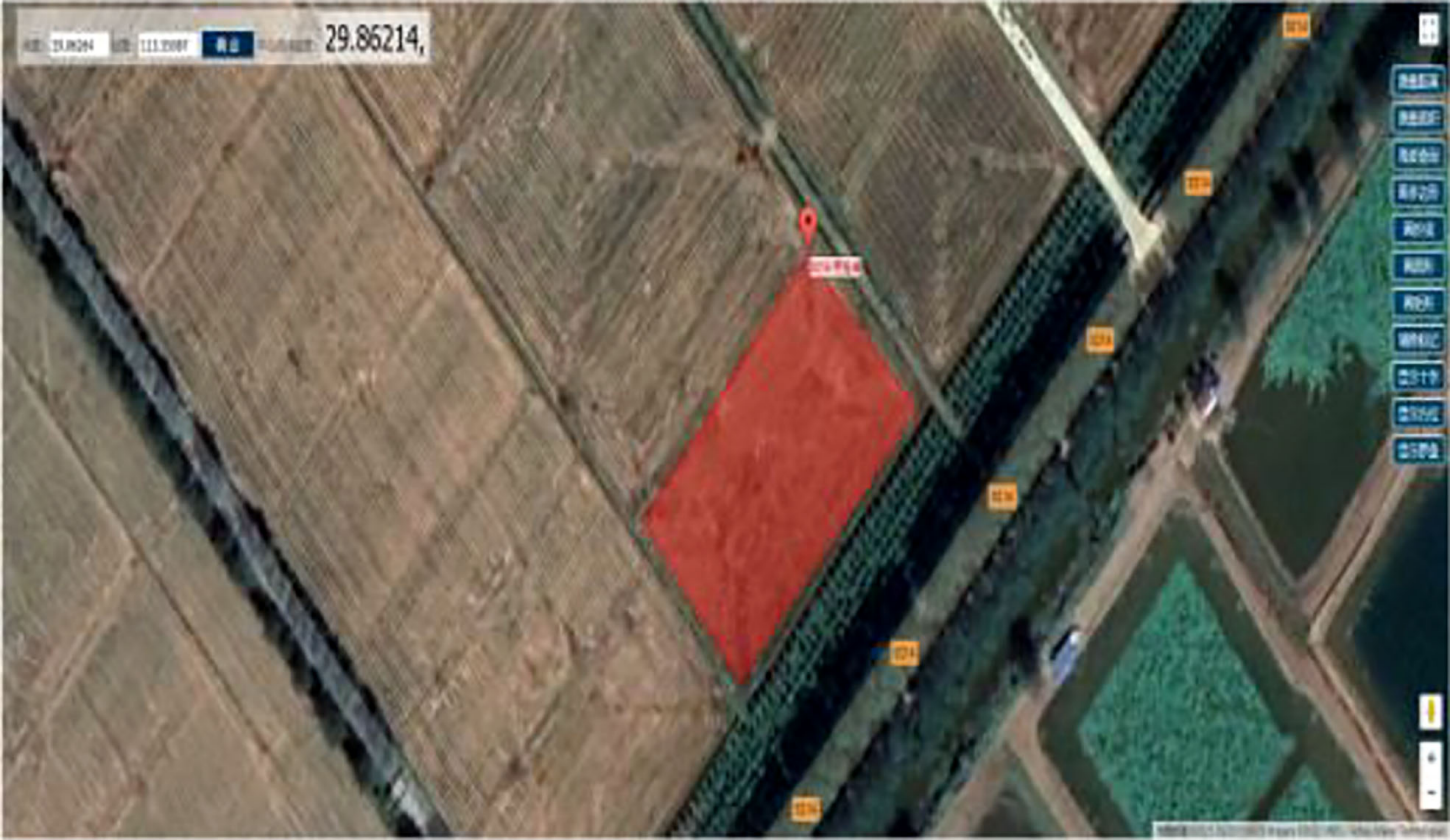
Satellite picture of regular field block.

From the satellite map, we can see the rectangular detour trajectory left by the harvester after the traditional harvesting method is completed, as shown in **Figure 8**. The area of the field was obtained as 3,520 m^2^ using the area measurement function of the satellite map, the long side of the field was 80 m, and the wide side was 48 m. The machine parameters were referenced to the dual-channel feed ratoon rice first-season harvester.

**Figure 9:**
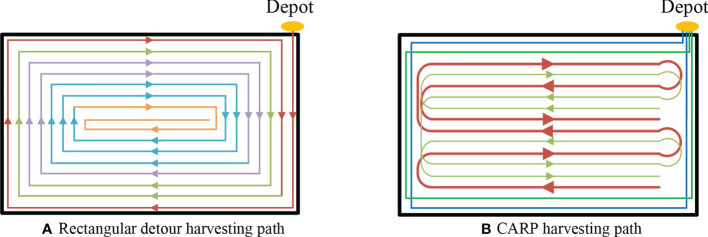
Path results under different path planning methods within a regular field block **(A)**. Rectangular detour **(B)**. CARP harvesting path harvesting path.

Based on the above test conditions, the path planning results from the rectangular detour harvesting method are shown in **Figure 9A**. In the rectangular detour harvesting path, the number of turns in one route gradually increases as the distance from the field boundary increases. Meanwhile, it is more likely for the harvester to be far away from the unloading point of grain storage full situation, finally resulting in greater grain loss in the grain unloading process.

Based on the path planning method proposed in this article, we obtain the set of crop rows *T*=[0, 1, 2, ⋯, 15] the set of stacking points *N*=[0, 1, 2, ⋯, 31] the width of the turning area at the head *W*_*t*_=6 the set of remaining crop rows *T*_1_=[2, 3, ⋯, 13] and the set of stacking points *N*_1_=[4, 5, ⋯, 27] After the harvesting of the turning area is completed, the CARP problem is solved by establishing the weight of grain between stacking points as the demand and the turning distance between stacking points as the cost consumption. The routing result of the row to be harvested is *S*_1_=[13→10→8→11→9→12] *S*_2_=[6→3→5→2→4→7] btained after solving using the improved GA, and the visualization result of the driving path is shown in **Figure 9B**. The number of grains unloading, the number of turns, the length of the harvester travel path, and the crushed area under the two harvesting methods were compared. Results are shown in **Table 5**.

**Table 5:** Comparison of different harvesting methods for regular field.

Comparative indicators	Rectangular detour harvesting	CARP	Improvement
Tours	5	4	20.00%
Turns	31	20	35.48%
Length (m)	2,297	1,758	23.46%
Crushed area (m^2^)	1,837.6	1,322.4	28.03%

Analysis of **Table 5** shows that the harvest path planning method proposed in this article has better performance in terms of the number of grains unloading, the number of turns in the field, and the length of the path traveled by the harvester and the area crushed. In particular, the number of turns was reduced by 35%, the total length of the harvester’s travel path was reduced by about 23%, and the crushed area of the field was reduced by about 28%.

This harvest path planning method achieves optimization on the total crushed area by reusing the crushed trajectories in the boundary area, which is a very important progress and can effectively improve the yield of ratoon rice in the second season. The harvest path planning method proposed in this research can meet the low crushing requirements for the first harvest of ratoon rice.

### 3.2 Comparison of different path planning methods in irregular-shaped fields

**Figure 10:**
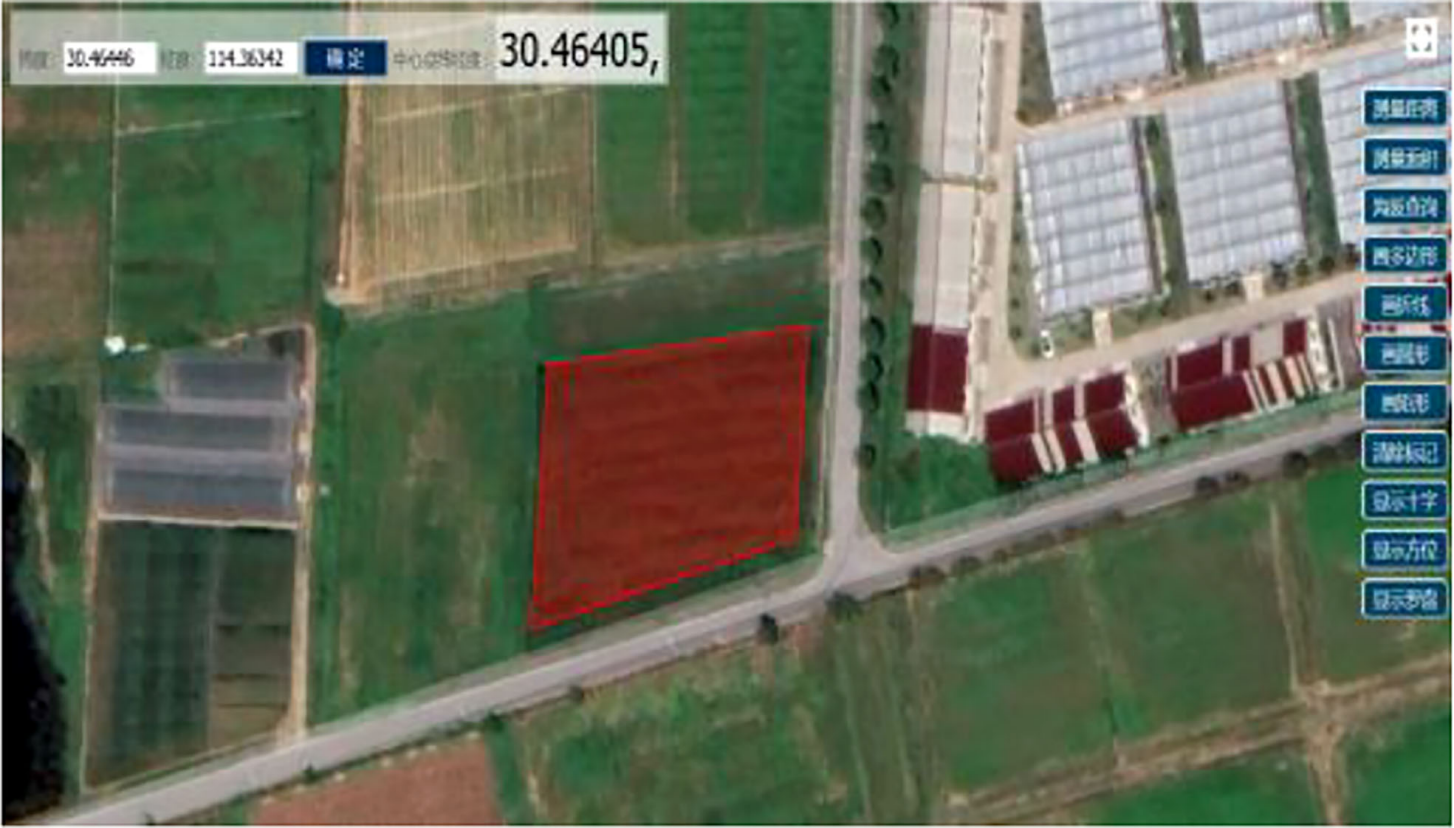
Satellite picture of irregular field block.

The irregular field was selected from a farmland in Hongshan District, Wuhan City, Hubei Province; the test plot profile information is shown in **Table 6**, and the field geometry is shown in **Figure 10**. The area of the field was obtained by the satellite map measurement function as 2,110 m^2^, and the side lengths of the field were 58 m, 36 m, 58 m, and 44 m, respectively. The yield of this field was about 8,250.1 kg/hm^2^ due to different planting conditions, and the traditional harvesting method used in this field was foldback reciprocating harvesting with adjacent strips operating in a “bulb-type” turn. Machine parameters remain consistent with the previous test.

**Table 6:** Simulation test irregular field plot contour point information.

Point Name	Latitude (°)	Longitude (°)
0	30.46446	114.36342
1	30.46419	114.36272
2	30.46458	114.36284
3	30.46459	114.36335

**Figure 11:**
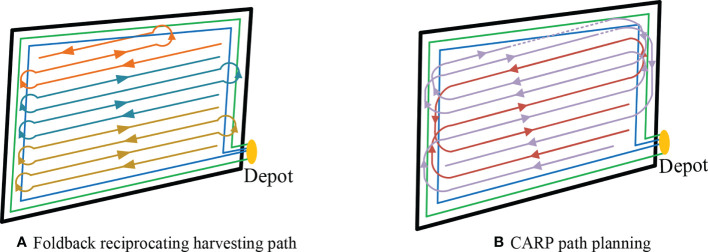
Path results under different path planning methods within an irregular field block **(A)**. Foldback **(B)**. CARP path planning reciprocating harvesting path.

The simplified field profile and the location of grain collection points and the foldback reciprocating harvesting route with the “bulb-type” turning strategy are shown in **Figure 11A**. After using the harvest path planning method proposed in this research, the harvester harvesting path was obtained as shown in **Figure 11B**. Data results were compared with the conventional foldback reciprocating harvest and are shown in **Table 7**.

**Table 7:** Comparison of different harvesting methods for irregular field.

Comparative indicators	Foldback reciprocating harvesting	CARP	Improvement
Tours	5	4	20.00%
Turns	27	23	14.81%
Length (m)	1,335	1,244	6.81%
Crushed area (m^2^)	818.3	726.6	11.21%

Both foldback reciprocating harvesting and the path planning method proposed in this research need to process the turning area at the head of the field. Under the same processing conditions, the differences between the two planning methods are mainly reflected in the scheduling between operating crop rows and the bin-full unloading link during the harvesting process.

According to the analysis in **Table 7**, it can be seen that the harvest path planning method proposed in this paper has a certain optimization effect on the number of grains unloading, the number of turns, the driving path length, and the crushing area compared with the foldback reciprocating harvest, among which the driving path length and the crushing area are reduced by 6.81% and 11.21% respectively.

The optimization degree of the two is different because compared with the single Ω-type turning strategy in cattle farming reciprocating harvest, the headland turning strategy used in this paper can make more full use of the harvester driving track generated in the headland turning area, effectively reduce the crushing area, and meet the requirements of the harvester driving path for the first harvest of reclaimed rice.

The difference in the degree of optimization between the two is due to the fact that the field-head turning strategy used in this paper makes fuller use of the harvester travel trajectory already generated in the field-head turning area compared to the single Ω-type turning strategy in foldback reciprocating harvesting, which can effectively reduce the crushing area and meet the requirements of the driving path of the harvester for the first-season harvest of ratoon rice.

### 3.3 Effects of harvester parameters

There are many factors that affect the harvesting of ratoon rice in the first season, including the parameters of the implements and the specific environment of the field. For example, when the width of the cutting table is large enough, once travel can complete the harvest of the entire field, which will undoubtedly bring about smaller crushing losses, but limited by the power of the machine and grain bin capacity and other restrictions, the width of the cutting table can only be increased within the adjustable range. In addition, the grain storage capacity of the grain bin of the harvester and the turning radius also have an impact on the effect of path planning; thus, the effect of these factors is explored using the length of the path travelled by the harvester and the crushed area after harvesting is completed as the measurement index.

For calculation and testing convenience, the simulation tests were conducted in rectangular fields with grain collection points located at the corners of the fields.

The rectangular detour path and the foldback reciprocating path in the traditional harvesting method were used as the control group, and two levels of *W* = 3 and *W* = 6 were taken for the width of the cutting table, and three levels were taken for the volume of the grain bin and the turning radius. The test indexes included the number of grains unloading, the number of turns, the length of the driving path, and the crushed area caused by harvesting. The test was conducted in a rectangular field with a side length of 60 × 80 The grain yield was set at 10,500 kg/hm^2^ and the comparison results are shown in **Table 8**.

**Table 8:** Effect of different machine parameters on path planning results.

Planning method	Grain bin capacity (Q)	Turning radius (R)	W=3m				W=6m			
			Tours	Turns	Length(/m)	Crushed area (/m^2^)	Tours	Turns	Length(/m)	Crushed area (/m^2^)
Rectangular detour	800	3	7	49	2,708	1,655.2	7	29	2,093	908.7
		5	7	49	2,965	1,850.4	7	29	2,251	1,022.7
		7	7	49	3,227	2,050.0	7	29	2,409	1,138.9
	1,200	3	5	46	2,554	1,630.5	5	27	1,895	896.3
		5	5	46	2,796	1,809.7	5	27	2,043	1,002.2
		7	5	46	3,042	1,992.4	5	27	2,191	1,110.1
	2,000	3	3	43	2,431	1,618.2	3	23	1,351	871.6
		5	3	43	2,657	1,789.2	3	23	1,478	961.2
		7	3	43	2,888	1,963.6	3	23	1,605	1,052.6
Foldback reciprocating	800	3	8	33	3,170	1,369.8	7	21	1,848	677.0
		5	8	37	3,239	1,444.1	7	21	1,945	698.7
		7	8	37	3,489	1,504.6	7	21	2,077	714.1
	1,200	3	5	31	2,868	1,382.3	5	18	1,379	673.2
		5	5	34	3,021	1,466.8	5	20	1,854	708.7
		7	5	34	3,301	1,537.3	5	20	1,981	729.2
	2,000	3	3	28	2,396	1,388.6	3	13	1,054	669.4
		5	3	32	2,829	1,478.2	3	17	1,332	718.7
		7	3	32	3,106	1,553.7	3	17	1,450	744.3
CARP	800	3	8	29	2,366	1,333.0	7	14	1,428	684.8
		5	7	28	2,539	1,368.9	7	16	1,413	686.2
		7	7	29	2,779	1,410.6	7	16	1,583	702.9
	1,200	3	5	26	2,310	1,331.9	6	14	1,285	664.9
		5	5	27	2,435	1,366.0	5	14	1,357	684.1
		7	5	27	2,717	1,406.0	5	14	1,456	698.9
	2,000	3	4	25	2,288	1,329.4	4	12	1,144	663.4
		5	3	25	2,288	1,364.7	3	13	1,330	681.9
		7	3	26	2,497	1,402.5	3	13	1,321	698.4

The path planning results with different harvester parameters are shown in **Table 8**. When the width of the cutting table is increased from *W* = 3 to *W* = 6, the total driving path length optimization rate of the harvester to complete the harvesting operation ranges from 22.71% to 56.01%, and the crushed area optimization rate ranges from 44.28% to 52.57%. Therefore, the width of the cutting table shows a positive correlation trend to the driving path length and crushing area optimization of the first-season mechanized harvesting of ratoon rice. When the width of the cutting table is large enough, the harvesting path length and the size of the crushed area obtained under different path planning methods are close.

With the same width of cutting table and capacity of grain bin, the driving path length and the resulting crushed area obtained under the three planning methods increase with the larger turning radius. However, the planning results obtained by the path planning method proposed in this article are better than those obtained by the traditional harvesting method, and the optimization margin gradually increases.

When the grain storage capacity of the harvester grain bin increases, the benefit of the paths obtained under the three planning methods increases accordingly. The harvester grain bin volume needs to be controlled within a reasonable range considering the crushing damage of the harvester on the rice stubble, at which time the path planning method proposed in this paper has an obvious optimization effect. For example, when *W* = 3 and *Q*=1,200 g, compared with the foldback reciprocating harvesting method, the length of the traveling path and the crushed area obtained by the CARP method are reduced by 18.80% and 6.44% on average under different turning radii.

In summary, the path planning method presented in this research can achieve a better path planning effect when the width of the harvester cutting table is small, the capacity of the grain bin is small, and the turning radius is large.

## 4 Conclusion

According to the special agronomic requirements of the first harvest of ratoon rice, this paper transformed the harvester operation path problem into the CARP, and used the improved GA in the selection and mutation link to solve it, so as to complete the harvesting path planning of the harvester.

The path planning method proposed in this article aims to divide the field into several operation rows according to the width of the harvester cutting table after processing the field for the head turn area. While assigning track sequence numbers to each operation crop row, the grain crops in the operation crop row are set to be stacked equally on the end points at both ends of the operation crop row. Giving arc constraints to two endpoints within the same operation row transforms the path planning problem of the harvester into a traversal order problem between stacked points. Therefore, the path planning of mechanized harvesting of ratoon rice in the first season converted to a same problem as CARP.

GA has good global search performance and high efficiency; thus, it is improved and used to solve the path planning problem. In order to verify that the path planning method proposed in this article is effective, specific planting plots in two regions were selected separately and compared with the corresponding traditional harvesting methods. The results show that the path planning method proposed in this research is more effective than the traditional harvesting method and can significantly reduce the crushed area during the harvesting process and increase the yield for the second season harvest of ratoon rice.

In the future, there are still many opportunities to carry out research regarding the related operational aspects of ratoon rice, such as localized harvester operation path planning based on the direction of collapse and the distribution of depot locations during the growth of ratoon rice, which, in turn, produces the best harvesting results. In addition, this study can provide a reference for other agricultural crops regarding operation paths. The CARP problem can be extended appropriately and solved by other intelligent algorithms with higher efficiency and better accuracy according to the specific operation types and operation conditions.

## Data availability statement

The raw data supporting the conclusions of this article will be made available by the authors, without undue reservation.

## Author contributions

GZ provided direction and key technical guidance for the study of this research. CJ completed the simulation testing of this research. QW made improvements in the study methodology. HL performed statistical analysis of the data. YZ provided guidance in the revision of the paper. JF wrote the first draft of this article. All authors contributed to the article and approved the submitted version.

## Funding

The work presented in this article was supported by the following project: the National Key Research and Development Program of China (Technological Innovation of Regenerated Rice Mechanization in the North of the Middle and Lower Reaches of the Yangtze River, 2017YFD0301404-05).

## Conflict of interest

The authors declare that the research was conducted in the absence of any commercial or financial relationships that could be construed as a potential conflict of interest.

## Publisher’s note

All claims expressed in this article are solely those of the authors and do not necessarily represent those of their affiliated organizations, or those of the publisher, the editors and the reviewers. Any product that may be evaluated in this article, or claim that may be made by its manufacturer, is not guaranteed or endorsed by the publisher.
